# Retinal Nerve Fiber Layer Rates of Change: Comparison of 2 OCT Devices

**DOI:** 10.1016/j.ogla.2025.02.005

**Published:** 2025-03-04

**Authors:** Vahid Mohammadzadeh, Erica Su, Iris Zhuang, Sajad Besharati, Justin Park, Andrea Yonge, Lynn Shi, Joseph Caprioli, Robert E. Weiss, Kouros Nouri-Mahdavi

**Affiliations:** 1 Glaucoma Division, Stein Eye Institute, David Geffen School of Medicine, University of California at Los Angeles, Los Angeles, California.; 2 Department of Biostatistics, Fielding School of Public Health, University of California Los Angeles, Los Angeles, California.

**Keywords:** Cirrus, Glaucoma progression, OCT, RNFL thickness, Spectralis

## Abstract

**Purpose:**

To compare retinal nerve fiber layer (RNFL) thickness rates of change and their variability between 2 commercial OCT devices.

**Design:**

Prospective cohort study.

**Participants:**

Ninety-four glaucoma eyes (94 patients) with central damage or moderate to advanced glaucoma with ≥ 2 years of follow-up and ≥ 4 pairs of OCT scans.

**Methods:**

A bivariate longitudinal Bayesian model was designed to compare inferences on RNFL rates of change from the 2 devices, both globally and in 12 clock hour sectors. Optic nerve OCT scans were acquired with Spectralis and Cirrus OCT devices in the same session. We inspected longitudinal RNFL profile plots from both OCT devices for all subjects across all sectors and globally.

**Main Outcome Measures:**

The rates of change, longitudinal variances, and proportions of significant negative and positive slopes (slope < 0 or > 0 μm/year and 1-sided *P* < 0.025, respectively) were compared between the devices.

**Results:**

The mean (standard deviation) baseline 24–2 visual field mean deviation and median (range) follow-up time were −8.2 (5.5) dB and 4.5 (2.2–6.7) years, respectively. The mean (95% credible interval [CrI]) estimated global baseline RNFL thickness for Spectralis and Cirrus OCTs were 61.5 (58.6–64.1) and 65.3 (63.2–67.4) μm, respectively. The global RNFL rates of change for Spectralis and Cirrus OCTs were −0.70 μm/year (95% CrI = −0.88 to −0.51 μm/year) and −0.45 μm/year (95% confidence interval = −0.63 to −0.27 μm/year) and were significantly faster for Spectralis compared to Cirrus OCT (difference = −0.24 μm/year, 95% CrI −0.45 to −0.04 μm/year, *P* < 0.001) as were sectoral rates in 5 out of 12 sectors. Higher proportions of significant negative RNFL rates of change were found with Spectralis OCT globally and in clock hour sectors 2 to 6 and 8 to 10 (corresponding to nasal, inferonasal, inferotemporal, and temporal regions). The proportions of significant positive rates of change were small (0%–3%) across sectors and similar between the devices.

**Conclusions:**

Spectralis OCT rates of RNFL change were faster compared to those from Cirrus OCT. Spectralis OCT detected a higher proportion of significant negative rates globally and in some sectors. OCT devices are not comparable regarding detection of change in eyes with central damage or moderate to advanced glaucoma.

**Financial Disclosure(s):**

Proprietary or commercial disclosure may be found in the Footnotes and Disclosures at the end of this article.

Glaucoma is a progressive optic neuropathy characterized by gradual damage of the retinal ganglion cells and their axons, the retinal nerve fiber layer (RNFL).^1^ The irreversible nature of such damage and the corresponding visual impairment makes early detection and monitoring of disease progression crucial in the care of patients with glaucoma.^2,3^ Structural damage may precede loss of visual function in glaucoma or it may be more easily detectable, highlighting the need for efficient imaging techniques to monitor disease progression.^4^ OCT is currently the standard tool for identifying structural changes in the optic nerve head, RNFL, or the macula in glaucoma.^5–9^

Several OCT devices are in widespread use in the United States. The 2 most frequently used devices are the Spectralis spectral-domain OCT (Heidelberg Engineering) and the Cirrus high-definition OCT (Carl Zeiss Meditce). Despite many similarities, the measurements from different OCT devices are not directly comparable. Differences in RNFL thickness measurements from different devices are largely attributable to the proprietary layer segmentation algorithms between the 2 devices.^10–12^ Consequently, it can be challenging for the clinician to monitor glaucoma progression over time if different devices are used on the same patient, and it is generally recommended that measurements from different machines not be used interchangeably for a given patient.^13,14^ There are no prior studies comparing the performance of different OCT devices regarding the detection of RNFL change over time. The aim of this study is to compare the RNFL rates of change (RoC) and their variance between 2 commercially available OCT devices in a cohort of eyes with central damage or moderate to severe glaucoma at baseline.

## Methods

### Patient Selection

A total of 158 patients were potentially eligible for this study, having had imaging with both the Cirrus and Spectralis OCT devices at ≥ 1 visits as part of the Advanced Glaucoma Progression Study, an ongoing longitudinal prospective study at the Stein Eye Institute. The more severely affected eye was chosen as the index eye if both eyes were eligible at the enrolment. The study was approved by the Human Research Protection Program at the University of California, Los Angeles. All patients provided consent at the time of enrolment, and all study procedures adhered to the tenets of the Declaration of Helsinki and the Health Insurance Portability and Accountability Act.

Inclusion criteria for the Advanced Glaucoma Progression Study included: (1) clinical diagnosis of primary open-angle glaucoma, primary angle-closure glaucoma, pseudoexfoliative glaucoma, and pigmentary glaucoma; (2) age between 40 and 80 years; (3) best-corrected visual acuity ≥ 20/50; (4) evidence of either central damage on 24–2 visual field (VF), defined as 2 or more points within the central 10° with *P* < 0.05 on the pattern deviation plot, or a VF mean deviation worse than −6 dB. Exclusion criteria were spherical refractive error exceeding 8 diopters (D) or ≥ 3 diopter cylinder, any significant retinal or neurological disease potentially affecting OCT measurements, and any ocular pathology (except cataract) at baseline. Study eyes underwent clinical exam and OCT imaging with both the Spectralis and Cirrus imaging at baseline and approximately every 6 months during the study.

All OCT images were reviewed to assess image quality (see Fig S1, available at www.ophthalmologyglaucoma.org). For the Cirrus OCT device, a total of 165 images out of 893 total images from 46 patients were excluded from this study due to image artifacts, segmentation errors, refixation artifacts involving the RNFL measurement circle, poor centration of the measurement circle, or signal strength < 6. An additional 16 patients were excluded for having < 4 acceptable quality scans with both OCT devices, resulting in a total of 94 patients.

### Spectralis OCT Imaging

The Spectralis RNFL scan consists of a circular scan 12° in diameter around the optic disc. The scan consists of 768 A-scans and is repeated 100 times to reduce speckle noise. The RNFL layer was segmented with the built-in Spectralis OCT software. The Spectralis OCT software allows the user to correct the segmentation manually. Based on a review by one of the authors (V.M.), 207 out of 1075 images (19%) were excluded due to low quality, uncorrectable segmentation error, schisis cavities, or epiretinal membrane. Retinal nerve fiber layer thickness measurements were exported to a personal computer. To allow for direct comparison to the clock hours of the corresponding Cirrus OCT images, the 768 pixels were divided into 12 corresponding clock hour sectors, with each clock hour consisting of 64 pixels. Clock hour 12 represents the superior location (right eye format), and the rest of the sectors from 1 to 11 represent corresponding sectors on the Cirrus OCT in a clockwise manner.

### Cirrus High Definition (HD)-OCT Imaging

Optic nerve imaging was performed with the Optic Disc Cube 200 × 200 algorithm, which centers the volume scan on the optic nerve head centroid and simultaneously scans the optic disc and peripapillary retina. The nominal dimensions of the scan in an emmetropic eye are 6 × 6 mm with 200 horizontal B-scans, each consisting of 200 A-scans. Layer segmentation was carried out with Cirrus OCT’s proprietary software.^15^

### Statistical Analyses

We inspected longitudinal RNFL profile plots from both OCT devices for all subjects across all sectors and globally. We applied a semiautomated algorithm to remove obvious outliers.^16^ Sectoral measurements from a given visit were removed for both Spectralis and Cirrus OCTs if they were identified as outliers on either device. A total of 99 (1.2%) pairs of observations were removed as outliers; 72 observations were identified as outliers on Cirrus only, 25 on Spectralis only, and 2 on both.

We analyzed global RNFL measurements and RNFL measurements for each sector separately using a bivariate Bayesian hierarchical model with random intercepts, random RoCs, random residual standard deviations (SDs), and correlated residuals. This model simultaneously models the RNFL RoCs for Cirrus and Spectralis OCTs, allowing direct comparison of the RNFL intercepts, RoC residuals, and residual SDs between the 2 devices. The correlated random intercepts, RoCs, and residual SDs provide a means to explore the associations between the RNFL measurements from the 2 devices.

We summarized the population intercepts, RoCs, and residual SDs with posterior means and 95% credible intervals (CrIs) at each sector and globally. We examined the differences between the intercepts and RoCs (Spectralis – Cirrus) and ratios of the residual SDs (Spectralis/Cirrus). We calculated the proportion of significantly negative RoCs and significantly positive RoCs globally and at each sector. We declared a RoC or difference in RoCs as significantly negative or positive if the upper or lower limit of the 95% CrI was less than or greater than 0, respectively. We declared a ratio as significantly smaller or larger if the upper or lower limit of the 95% CrI was less than or greater than 1, respectively. We compared the proportion of significantly negative and positive RoCs between devices using Bayesian McNemar tests at each sector and globally. Analyses were performed using R version 4.3.1 and Bayesian models were implemented with the R package NIMBLE.^17–19^

## Results

**Figure 2.**
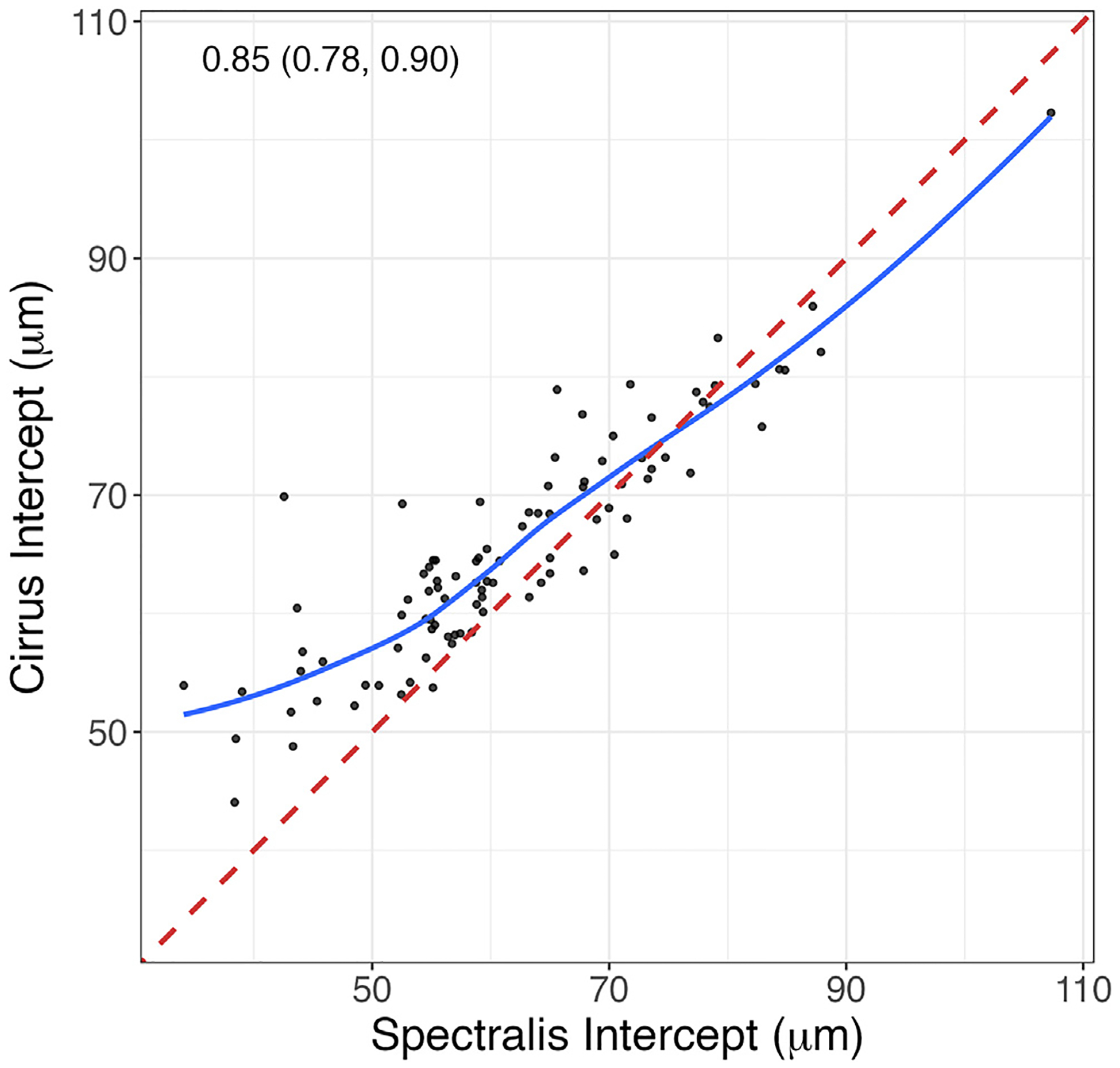
Scatterplot of Cirrus OCT global RNFL intercepts (baseline thickness measurements in μm) against those from Spectralis OCT. The posterior mean (95% credible interval) between-device correlation was 0.85 (0.78–0.90). The red dashed line is the line of unity and the blue curve is the lowess fit. RNFL = retinal nerve fiber layer.

Final data analyses were performed on 94 eyes of 94 patients. The mean (SD) age and baseline 24–2 VF mean deviation was 67.1 (8.2) years and −8.2 (5.5) dB, respectively. The median (range) follow-up time was 4.5 (2.2–6.7) years. Table 1 provides the demographic and clinical characteristics of the study cohort. The mean (95% CrI) estimated global RNFL thickness for Spectralis and Cirrus OCT was 61.5 (58.6–64.1) μm and 65.3 (63.2–67.4) μm, respectively, and was significantly lower for Spectralis OCT compared to Cirrus OCT (difference = −3.85 μm, 95% CrI −5.28 to −2.42, *P* < 0.001). Figure 2 shows a scatterplot of the posterior means of the estimated global RNFL thickness from Cirrus OCT against those of Spectralis OCT. Figure S3 (available at www.ophthalmologyglaucoma.org) provides scatterplots of Cirrus OCT against Spectralis OCT intercepts for each of the 12 clock hour sectors. The correlations between intercepts were generally high (0.67–0.93) except in sectors 3 and 4 with correlation means of 0.34 and 0.48, respectively (Table S2, available at www.ophthalmologyglaucoma.org). Globally, and in most sectors, RNFL thickness measurements from Spectralis tended to have higher values on the higher end and lower values on the lower end of the measurements, an indication of a potentially higher dynamic range for Spectralis OCT compared to Cirrus OCT.

**Figure 4.**
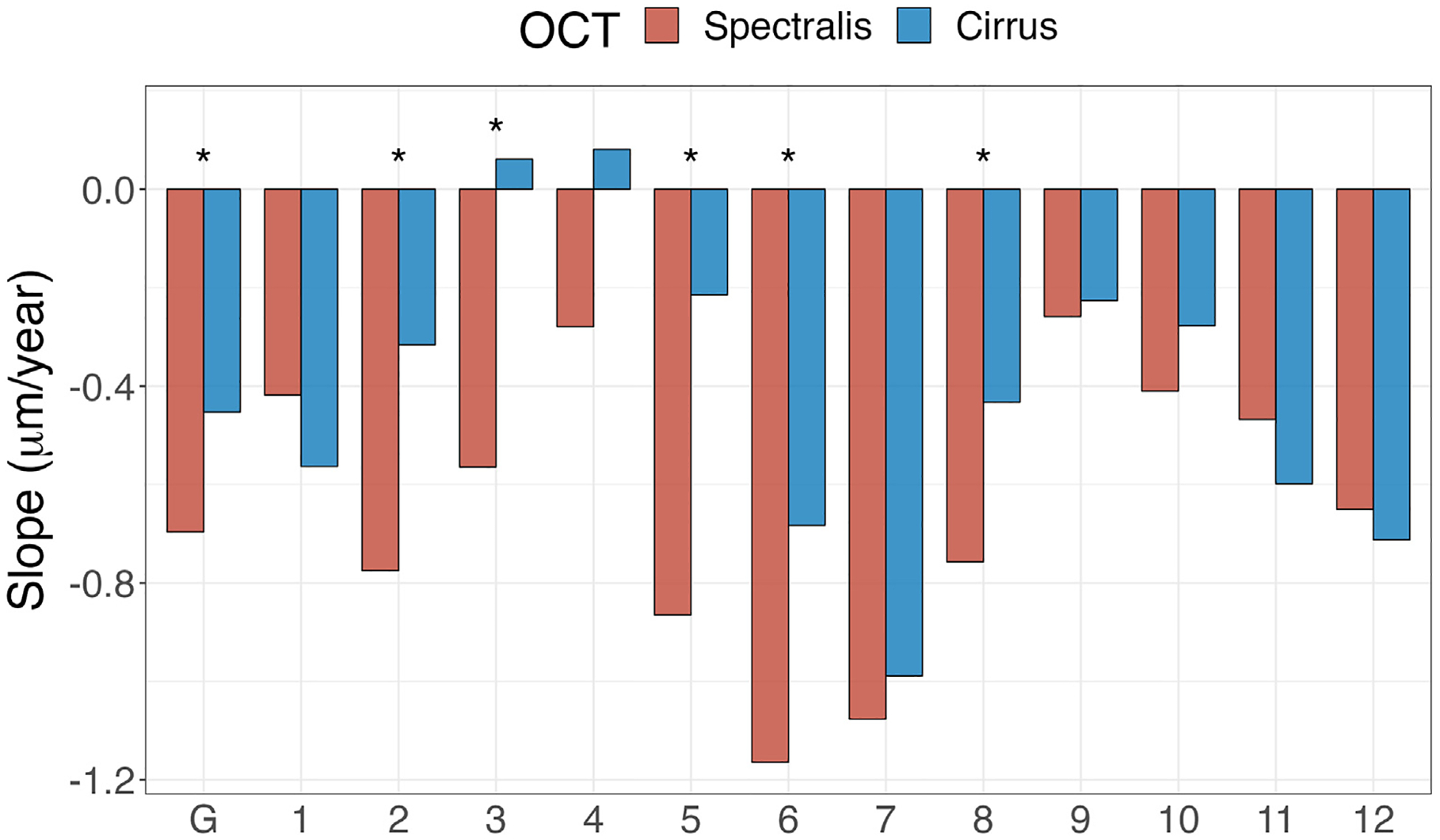
Bar plot of posterior mean population RNFL rates of change (μm/year) for Spectralis and Cirrus OCT devices globally and in 12 clock hour sectors. Asterisks indicate sectors where Spectralis RNFL rate of change is significantly faster compared to those of Cirrus OCT. RNFL = retinal nerve fiber layer.

Table 3 lists posterior means of the population RoCs globally and for the 12 clock hour sectors for the Spectralis and Cirrus OCT devices. The global RNFL RoCs for Spectralis and Cirrus OCTs were −0.70 μm/year (95% CrI = −0.88 to −0.51 μm/year) and −0.45 μm/year (95% CrI = −0.63 to −0.27 μm/year), respectively, and were significantly faster for Spectralis OCT compared to Cirrus OCT (difference = −0.24 [−0.45 to −0.04] μm/year). The population RoCs were significantly faster for Spectralis OCT compared to Cirrus OCT in clock hour sectors 2, 3, 5, 6, and 8. Figure 4 displays a bar plot of posterior mean RNFL RoCs globally and for each clock hour sector for the 2 OCT devices.

**Figure 5.**
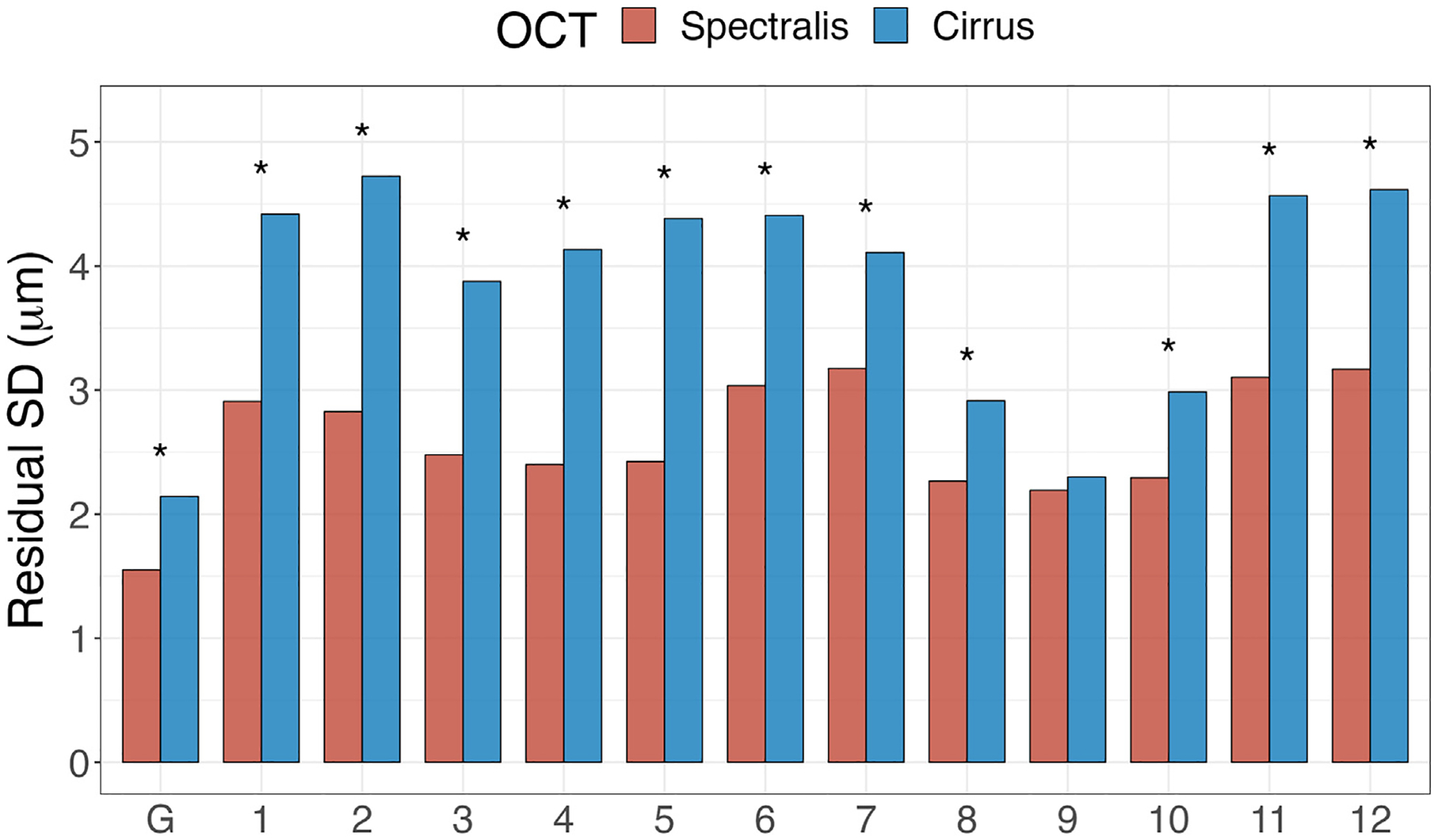
Posterior mean residual SDs (μm) for retinal nerve fiber layer measurements from Spectralis and Cirrus OCT devices; asterisks indicate significantly smaller mean residual SDs for Spectralis OCT. SD = standard deviation.

**Figure 6.**
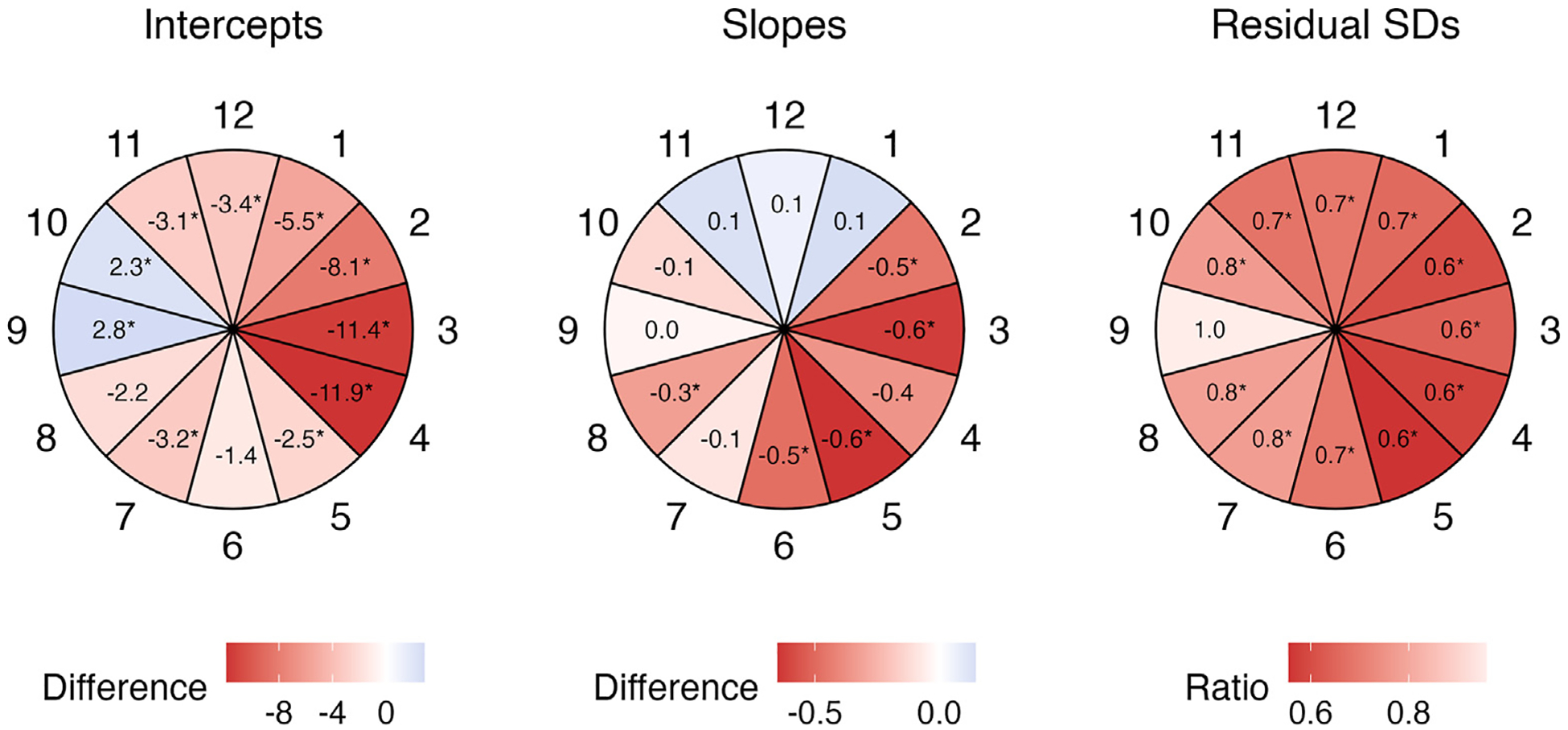
Difference between the RNFL rates of change (μm/year) and the estimated RNFL intercepts (μm) for Spectralis and Cirrus OCT devices (Spectralis – Cirrus) and the ratio of residual SDs (Spectralis/Cirrus) in 12 clock hour sectors. For the intercepts, the blue color indicates that Spectralis OCT has a thicker baseline RNFL thickness and pink/red colors denote that Cirrus OCT has a thicker baseline thickness, with darker colors indicating greater differences; asterisks flag sectors with significant differences between the 2 OCT devices. For rates of change, the blue color means that Cirrus OCT has faster RNFL rates of change while pink/red colors mean that Spectralis OCT has faster RNFL rates; asterisks flag sectors with significant differences between the 2 devices. For the residual SDs, pink/red colors mean that Spectralis OCT has lower average residual SDs compared to Cirrus OCT. Spectralis OCT had significantly lower residual variance in all sectors except sector 9. RNFL = retinal nerve fiber layer; SD = standard deviation.

Figure 5 is a bar chart of posterior means of the average residual variance for Spectralis and Cirrus OCT devices globally and for the 12 clock hour sectors. The average residual variance was significantly smaller for Spectralis OCT compared to Cirrus OCT globally and in 11 clock hour sectors, excluding only sector 9. Table S4 (available at www.ophthalmologyglaucoma.org) gives the posterior mean (95% CrI) of the average residual RNFL variances globally and at 12 sectors for Spectralis and Cirrus OCTs. Figure 6 demonstrates heat maps of the difference between RNFL intercepts (left panel) and RoCs (middle panel) from Spectralis and Cirrus OCT devices (Spectralis – Cirrus) and the ratio of residual SDs (Spectralis/Cirrus) (right panel). Spectralis OCT had significantly thinner baseline thickness measurements (intercepts) in 8 sectors (11-, 12-, 1-, 2-, 3-, 4-, 5-, and 7-o’clock sectors), significantly thicker baseline thickness in 2 sectors (9- and 10-o’clock sectors), and faster population RoCs for 5 of 12 sectors (2-, 3-, 5-, 6-, and 8-o’clock sectors), and also showed lower residual SD (variability) compared to Cirrus OCT in 11 of 12 sectors.

**Figure 7.**
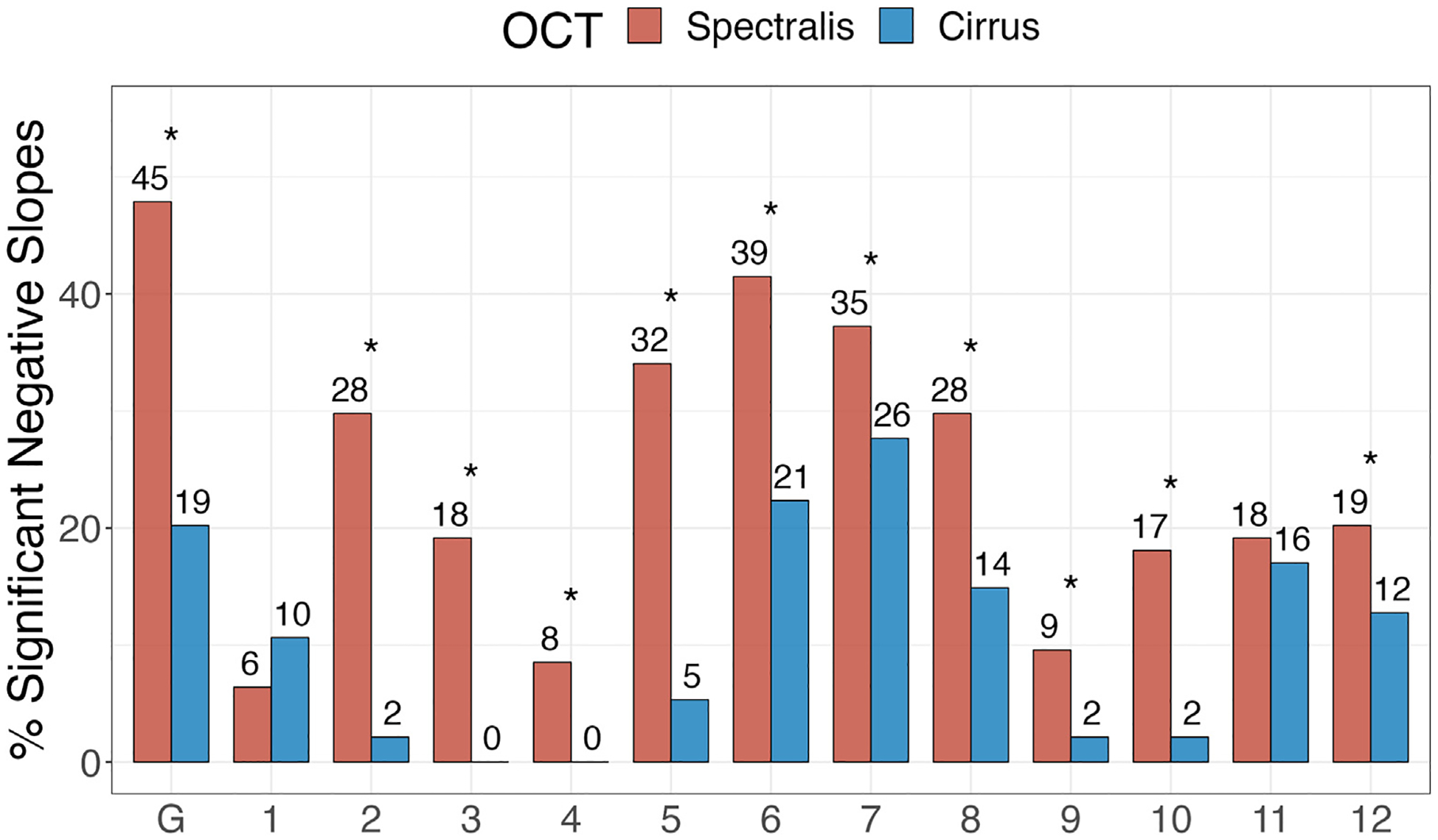
Percentage of significantly negative retinal nerve fiber layer rates of change globally and at the 12 clock hour sectors for the Spectralis and Cirrus OCT devices. The number of eyes with significant rates of change is shown on top of each bar.

**Figure 8.**
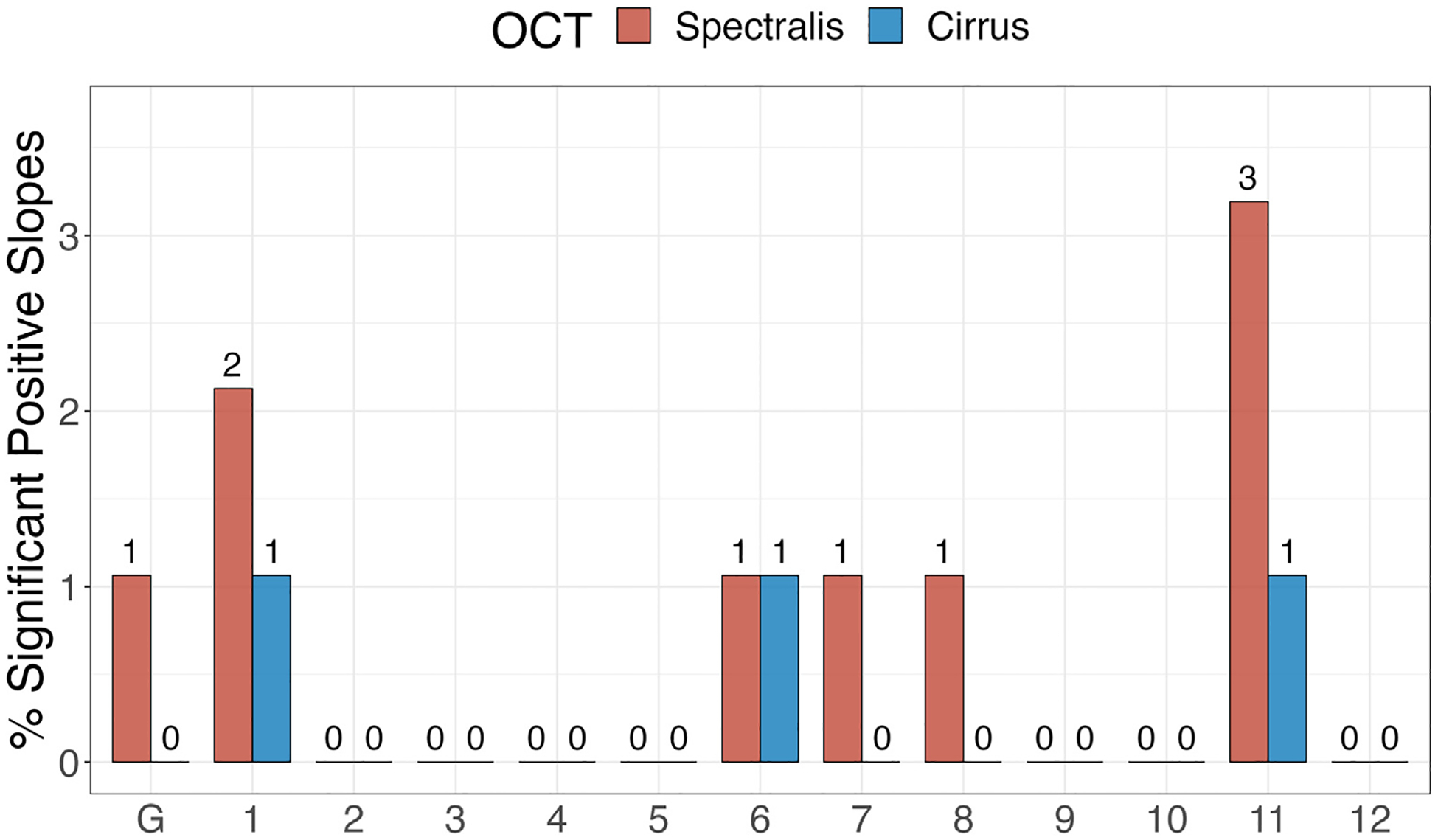
Percentage of significant retinal nerve fiber layer positive rates of change globally and at the 12 clock hour sectors for the Spectralis and Cirrus OCT devices. The number of eyes with significant rates of change is shown on top of each bar.

Figure 7 provides the proportions of significantly negative RoCs globally and in the 12 clock hour sectors for the 2 OCT devices. A higher proportion of significantly negative RNFL RoCs was observed for Spectralis OCT compared to Cirrus OCT globally and in clock hour sectors 2 through 6 and 8 through 10 corresponding to nasal, inferonasal, inferotemporal, and temporal regions. The proportions of significantly positive RoCs were small and not significantly different between the 2 OCT devices (Fig 8).

## Discussion

Several clinical studies have used trend analyses on structural measures to assess glaucoma progression, with a statistically significant negative RoC being considered as evidence of disease deterioration. We designed a bivariate longitudinal Bayesian hierarchical model to compare rates of RNFL change based on data from 2 commercially available OCT devices in eyes with central damage or moderate to advanced glaucoma. We found that the rates of RNFL change were significantly faster globally and for nearly half of the 12 clock hour sectors when measured with Spectralis OCT compared with Cirrus OCT. The average residual variance, an outcome measure reflecting the magnitude of the noise in longitudinal RNFL measurements, was lower for Spectralis OCT compared to Cirrus OCT. The proportion of significantly negative RoCs, an indicator of clinically relevant structural worsening, was significantly higher globally and in 8 of the 12 clock hour sectors in the nasal, inferonasal, inferotemporal, and temporal regions when measured with Spectralis OCT.

The utility of OCT RNFL measurements for glaucoma detection and monitoring of disease progression has been evaluated in many previous studies.^20–26^ Retinal nerve fiber layer measurements demonstrate very good reproducibility, an important feature related to the detection of change^20,27^; they also show a good correlation with VF measurements.^28–32^ A major limitation of any structural measure, including RNFL, is the measurement floor with advancing glaucoma damage.^33^

A very slow attrition of RNFL thickness can be seen in healthy eyes, which is attributed to aging. The average rates of global RNFL change in healthy eyes range from −0.07 μm/year to −0.52 μm/year.^34–37^ Rates of RNFL change in glaucomatous eyes are expected to be higher but can vary markedly based on the type of device being used and the severity of the disease at baseline. With advancing damage and lower baseline RNFL thickness, rates of RNFL thinning tend to decrease as measurements approach the measurement floor. Several studies compared the diagnostic accuracy between Spectralis and Cirrus OCT.^10,38^ However, no study has previously compared their performance regarding the detection of RNFL change. Prior studies found poor agreement between structural progression at the level of RNFL and VF progression^39^; hence, functional findings cannot be easily used to compare the performance of different OCT devices with regard to the detection of change.

In our study, Spectralis RNFL trend analyses were able to detect glaucoma structural change more efficiently than the RNFL trend analyses of Cirrus OCT in eyes with central damage or moderate to advanced glaucoma. Global RNFL slopes and RNFL slopes at 3 nasal sectors and inferior and inferotemporal sectors were significantly faster for Spectralis OCT. Glaucoma damage tends to start in the superotemporal and inferotemporal regions^40^; because our study population mostly consisted of patients with moderate to advanced glaucoma, we expected to see faster slopes in sectors that were not close to the RNFL thickness measurement floor (including nasal sectors). Therefore, in patients with more advanced glaucoma, RNFL RoCs in such sectors could be used for monitoring structural glaucoma progression over time.

The Spectralis OCT software uses repeated image acquisition to enhance scan quality with single circular RNFL measurements repeated 100 times, thus creating higher-resolution single B-scan images. While both devices use automated segmentation of the RNFL layer boundaries, Spectralis OCT software allows for manual correction of segmentation errors by the user. This has been shown, in previous studies, to improve the RNFL thickness measurements and could partially explain the higher detection rates of the Spectralis OCT in our study.^41^ Another important factor that could explain the difference in RNFL rates between the 2 OCT devices is the higher dynamic range that was observed with Spectralis OCT measurements (Fig S3).

The proportion of significantly negative RoCs was used as a proxy for assessing the OCT device performance for the detection of structural glaucoma progression (Fig 7). For most of the clock hour sectors and globally, Spectralis OCT identified a significantly higher proportion of significantly negative RoCs. In all clock hour sectors,^40,42^ the amount of noise seemed to be higher for Cirrus OCT compared to Spectralis OCT (Fig 5); therefore, Spectralis OCT would be expected to perform better in detecting the actual signal change.

The Cirrus HD-OCT summarizes RNFL thickness measurements in clock sectors whereas Spectralis OCT averages the RNFL thickness from 768 A-scans on the measurement circle in 6 unequal sectors. To match the RNFL measurements between the 2 devices, we exported and created clock hour sectors for the Spectralis RNFL thickness measurements. The Spectralis RNFL measurements are estimated to be within 1 decimal while Cirrus OCT RNFL measurements are rounded to the nearest integer. The reported results in this manuscript are derived from nonrounded Spectralis RNFL measurements. We also repeated the analyses after rounding Spectralis thickness measurements; the results from the second set of analyses were similar to those from the nonrounded RNFL thickness measurements (results not shown). On average, the rounded Spectralis measurements increased the global residual SDs from 0% to 2% compared to the nonrounded thickness measurements in the 12 sectors and globally.

We designed a joint bivariate Bayesian model for estimating and comparing the rates of RNFL OCT between the devices. This model offers a robust approach for analyzing 2 related longitudinally measured variables simultaneously by leveraging their correlation within a Bayesian framework.^43^ Unlike separate models applied to correlated variables, this approach explicitly accounts for the relationship between correlated variables leading to more accurate and reliable inferences.^44^ By integrating prior knowledge regarding observed data, the model creates a joint probability distribution that not only improves predictive accuracy but also provides a comprehensive quantification of uncertainty through CrIs, which helps compare measurement accuracy between parameters of interest (between 2 OCT devices in our study). Its flexibility in accommodating complex data structures and prior information makes it particularly advantageous in situations where interdependence of outcome measures needs to be accounted for. In our study, there is an inherent correlation of RNFL thickness measured with different OCT devices and hence, this model is an appropriate way to compare the RoCs between OCT devices. These features make the joint bivariate Bayesian model the superior choice for comparison of structural RoCs and for drawing meaningful conclusions.

Our study’s limitations need to be considered. While poor-quality images from either device led to the exclusion of the corresponding measurements for both devices, some minor issues persisted. These issues include minor localized segmentation issues, vitreous opacities affecting local RNFL measurements, slight displacement of measurement circle due to inaccurate Bruch membrane opening delineation, occasional subtle refixation artifacts, and localized RNFL changes caused by vitreomacular traction, all of which could lead to increased noise or outliers. Although the Spectralis software allows users to correct segmentation errors, Cirrus OCT measurements cannot be altered or corrected.

In conclusion, we found a significant difference in RNFL RoCs between Spectralis and Cirrus OCT devices in the study cohort, which consisted of eyes with central damage or moderate to severe glaucoma. Overall, Spectralis OCT detected faster RoCs globally and in some sectors and identified a higher proportion of significantly negative RoCs in this cohort. Clinicians and researchers need to be aware that OCT devices are not comparable with regard to the detection of change in glaucoma eyes. It is also likely that using thickness measurements from different OCT devices during the follow-up period would lead to increased measurement noise and decrease the ability to detect RNFL change over time.

## Supplementary Material

1

2

3

4

Supplemental material available at www.ophthalmologyglaucoma.org.

## Figures and Tables

**Table 1.** Demographic and Clinical Characteristics of the Study Cohort

	Number
No. of eyes (patients)	94 (94)
Mean (SD) age, yrs	67.1 (8.2)
Sex, female/male, n (%)	57/38 (60.0/40.0)
Median no. of OCT pairs (range)	7 (4–13)
Median (range) follow-up time, yrs	4.5 (2.2–6.7)
Mean (SD) intraocular pressure, mmHg	12.7 (4.0)
Mean (SD) baseline 24–2 MD, dB	−8.2 (5.5)
Mean (SD) global RNFL thickness at baseline, Spectralis OCT, μm	61.5 (13.3)
Mean (SD) global RNFL thickness, Cirrus OCT, μm	65.3 (10.5)

MD = mean deviation; RNFL = retinal nerve fiber layer; SD = standard deviation.

**Table 3.** The Posterior Mean and 95% Credible Intervals for Retinal Nerve Fiber Layer Rates of Change (μm/Year) for Cirrus and Spectralis OCT Devices

	Spectralis			Cirrus			Difference (Spectralis — Cirrus)		
Sector	*Mean*	*2.5%*	*97.5%*	*Mean*	*2.5%*	*97.5%*	*Mean*	*2.5%*	*97.5%*
Global	−0.70	−0.88	−0.51	−0.45	−0.63	−0.27	−0.24	−0.45	−0.04
1	−0.42	−0.67	−0.16	−0.56	−0.92	−0.20	0.14	−0.20	0.50
2	−0.77	−1.04	−0.52	−0.32	−0.64	0.01	−0.46	−0.83	−0.10
3	−0.56	−0.78	−0.35	0.06	−0.21	0.34	−0.63	−0.95	−0.29
4	−0.28	−0.51	−0.03	0.08	−0.19	0.35	−0.36	−0.73	0.01
5	−0.86	−1.16	−0.58	−0.21	−0.56	0.13	−0.65	−0.99	−0.31
6	−1.16	−1.54	−0.80	−0.68	−1.11	−0.26	−0.48	−0.85	−0.12
7	−1.08	−1.40	−0.78	−0.99	−1.37	−0.61	−0.09	−0.48	0.30
8	−0.76	−1.01	−0.51	−0.43	−0.66	−0.21	−0.32	−0.63	−0.02
9	−0.26	−0.45	−0.07	−0.23	−0.42	−0.04	−0.03	−0.28	0.22
10	−0.41	−0.64	−0.17	−0.28	−0.51	−0.05	−0.13	−0.43	0.17
11	−0.47	−0.79	−0.14	−0.60	−0.98	−0.22	0.13	−0.22	0.49
12	−0.65	−0.93	−0.39	−0.71	−1.05	−0.37	0.06	−0.27	0.40

The difference between Spectralis and Cirrus rates of change and the corresponding 95% credible intervals are provided in the last 3 columns. Spectralis OCT had significantly faster retinal nerve fiber layer rates of change than Cirrus OCT globally, as well as in clock hour sectors 2, 3, 5, 6, and 8.

## Abbreviations and Acronyms:

CrI: credible interval
HD: high definition
RNFL: retinal nerve fiber layer
RoC: rates of change
SD: standard deviation
VF: visual field
