# Supplementary material for: Retinal Nerve Fiber Layer Rates of Change: Comparison of 2 OCT Devices
Source: Ophthalmol Glaucoma. Author manuscript; Available in PMC 2026 May 25. (PMC13200281; doi:10.1016/j.ogla.2025.02.005)
Supplement: 1 [file NIHMS2172310-supplement-1.pdf]

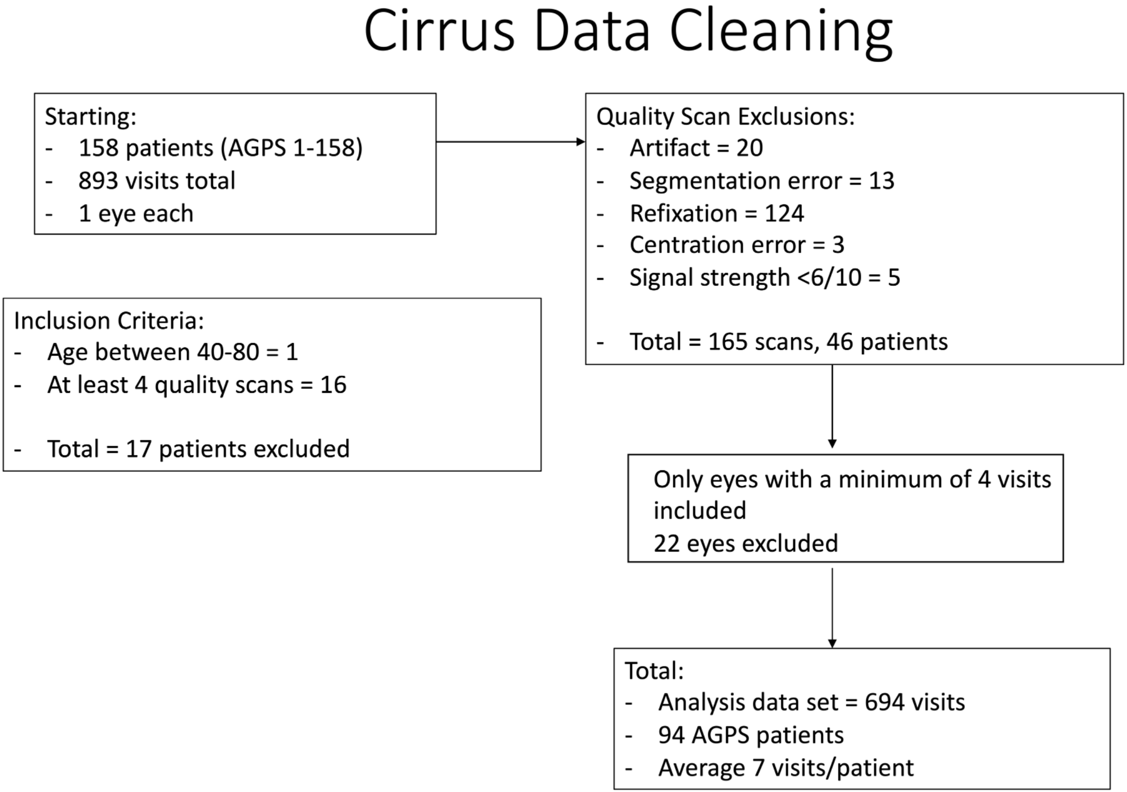

Supplementary Figure 1. Flow chart depicts the process of excluding poor quality images for the Cirrus OCT retinal nerve fiber layer measurements.
