# Supplementary material for: Retinal Nerve Fiber Layer Rates of Change: Comparison of 2 OCT Devices
Source: Ophthalmol Glaucoma. Author manuscript; Available in PMC 2026 May 25. (PMC13200281; doi:10.1016/j.ogla.2025.02.005)
Supplement: 2 [file NIHMS2172310-supplement-2.pdf]

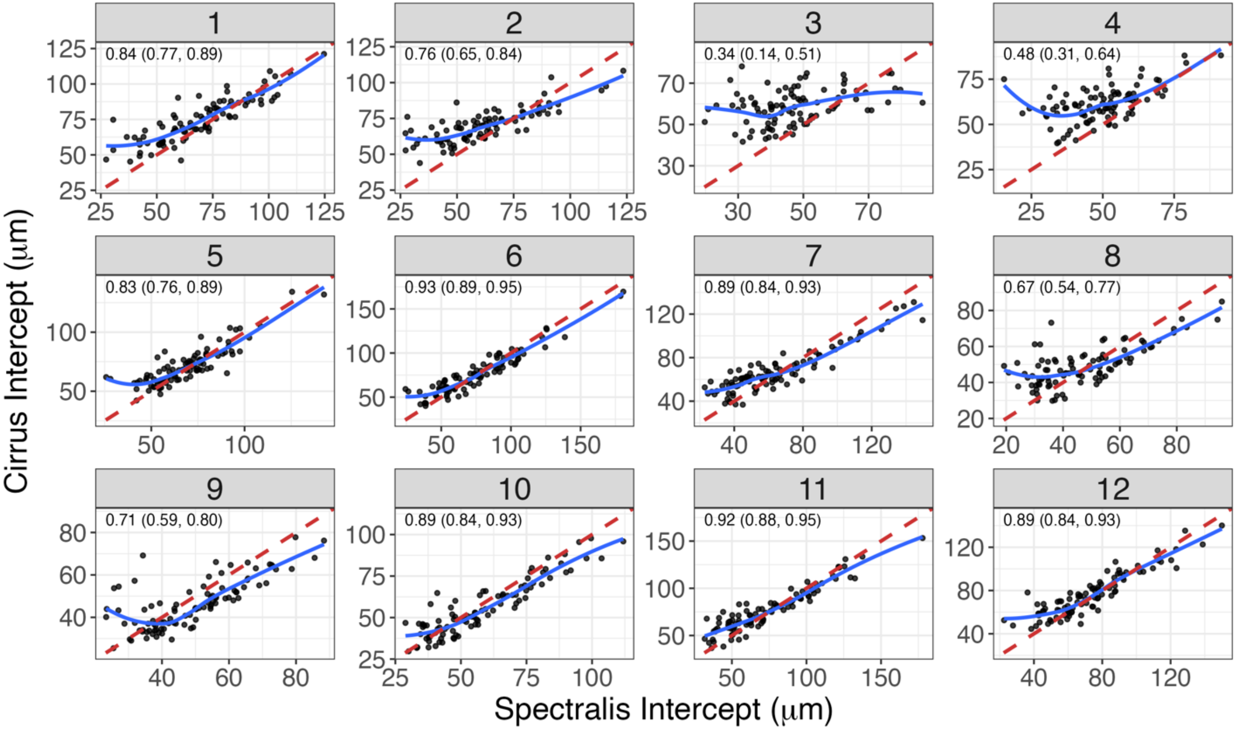

Supplementary Figure 3. Scatterplots of posterior means of the retinal nerve fiber layer intercepts (estimated baseline thickness measurements in μm) for Cirrus against Spectralis OCT at 12 clock-hour sectors. The posterior mean (95% credible interval) of the between-device correlation is shown on the top left of each scatterplot. The red dashed line represents the $x = y$ line, and the blue curve is the lowess fit.
