# Supplementary material for: Retinal Nerve Fiber Layer Rates of Change: Comparison of 2 OCT Devices
Source: Ophthalmol Glaucoma. Author manuscript; Available in PMC 2026 May 25. (PMC13200281; doi:10.1016/j.ogla.2025.02.005)
Supplement: 3 [file NIHMS2172310-supplement-3.pdf]

Supplementary Table 4. Posterior mean of residual standard deviations (μm) and 95% credible intervals for global and sectoral Spectralis and Cirrus retinal nerve fiber layer. The last 3 columns provide the posterior mean and 95% credible intervals of the ratio of Spectralis residual SD over Cirrus residual SD. A value less than 1 means that Spectralis data had smaller residual SDs on average. The values are significantly smaller for Spectralis OCT globally and in 11 of 12 clock-hour sectors except for sector 9.

	**Spectralis**			**Cirrus**			**Ratio (Spectralis / Cirrus)**		
**Sector**	**Mean**	**2.5%**	**97.5%**	**Mean**	**2.5%**	**97.5%**	**Mean**	**2.5%**	**97.5%**
Global	1.55	1.35	1.77	2.14	1.88	2.43	0.73	0.62	0.84
1	2.91	2.52	3.33	4.42	3.92	4.96	0.66	0.56	0.78
2	2.83	2.47	3.23	4.72	4.22	5.28	0.60	0.51	0.70
3	2.48	2.17	2.81	3.88	3.45	4.34	0.64	0.54	0.76
4	2.40	2.07	2.77	4.13	3.66	4.62	0.58	0.48	0.69
5	2.43	2.13	2.75	4.38	3.90	4.91	0.56	0.47	0.65
6	3.04	2.69	3.41	4.41	3.92	4.96	0.69	0.59	0.81
7	3.17	2.75	3.65	4.11	3.65	4.62	0.77	0.65	0.92
8	2.27	1.99	2.57	2.91	2.55	3.33	0.78	0.65	0.92
9	2.19	1.91	2.50	2.30	1.97	2.66	0.96	0.80	1.14
10	2.29	1.99	2.62	2.99	2.61	3.39	0.77	0.65	0.91
11	3.10	2.70	3.55	4.57	4.03	5.15	0.68	0.58	0.80
12	3.17	2.74	3.61	4.62	4.07	5.20	0.69	0.58	0.81
