# Supplementary material for: Retinal Nerve Fiber Layer Rates of Change: Comparison of 2 OCT Devices
Source: Ophthalmol Glaucoma. Author manuscript; Available in PMC 2026 May 25. (PMC13200281; doi:10.1016/j.ogla.2025.02.005)
Supplement: 4 [file NIHMS2172310-supplement-4.pdf]

Supplementary Table 2. Posterior mean and 95% credible intervals of the between-device correlations for retinal nerve fiber layer intercepts and slopes. The intercept correlations were generally higher than those for slopes. The correlations were significantly positive globally and in all sectors for intercepts and significantly positive globally and in only 6 sectors for slopes.

	**Intercepts**			**Slopes**		
**Sector**	**Mean**	**2.5%**	**97.5%**	**Mean**	**2.5%**	**97.5%**
Global	0.85	0.78	0.90	0.50	0.23	0.72
1	0.84	0.77	0.89	0.66	0.39	0.84
2	0.76	0.65	0.84	0.41	0.00	0.71
3	0.34	0.14	0.51	0.21	–0.21	0.57
4	0.48	0.31	0.64	–0.12	-0.53	0.35
5	0.83	0.76	0.89	0.68	0.42	0.84
6	0.93	0.89	0.95	0.81	0.66	0.91
7	0.89	0.84	0.93	0.63	0.33	0.83
8	0.67	0.54	0.77	0.33	–0.03	0.63
9	0.71	0.59	0.80	0.21	–0.16	0.54
10	0.89	0.84	0.93	0.11	–0.31	0.52
11	0.92	0.88	0.95	0.76	0.55	0.89
12	0.89	0.84	0.93	0.61	0.27	0.83
